# Secukinumab-Induced Inflammatory Bowel Disease in a Patient Treated for Chronic Plaque Psoriasis and Psoriatic Arthritis: A Case Report and Review of the Role of Novel Biologic Agents Targeting the p19 Subunit of IL-23

**DOI:** 10.1155/2020/9404505

**Published:** 2020-07-24

**Authors:** K. M. Darch, T. L. Holland, L. J. Spelman

**Affiliations:** Veracity Clinical Research, Woolloongabba, QLD 4102, Australia

## Abstract

Chronic plaque psoriasis and psoriatic arthritis are common autoimmune inflammatory conditions, often existing as comorbidities that have a significant impact on a patient's quality of life. The availability of a range of novel, targeted therapies for their treatment, most notably the availability of biologic agents, has revolutionised management of these conditions and allowed for significant improvements in patient outcomes. Secukinumab (Cosentyx), a fully-human monoclonal antibody that acts by selectively binding to and neutralising the proinflammatory cytokine IL-17A, is used widely for the treatment of both chronic plaque psoriasis and psoriatic arthritis. In this report, we discuss the case of a 54-year-old female with chronic plaque psoriasis and psoriatic arthritis, who experienced the onset of symptoms and was subsequently diagnosed with Crohn's disease during treatment with secukinumab. It was determined by the gastroenterologist that this may represent secukinumab-induced inflammatory bowel disease. We will review the current understanding of the role of IL-17 in inflammatory bowel disease and the important role of novel agents that target the p19 subunit of IL-23 which have shown significant promise in the management of not only chronic plaque psoriasis and psoriatic arthritis, but also inflammatory bowel disease itself.

## 1. Introduction

Chronic plaque psoriasis is a common, autoimmune condition of the skin, affecting 1-2% of the population [1]. Psoriatic arthritis, an inflammatory and potentially destructive condition of the joints, has a population prevalence of 1% and is seen in up to 30% of patients with chronic plaque psoriasis, most commonly associated with the presence of psoriatic nail disease or psoriasis of the scalp [2, 3]. These conditions, particularly when experienced together, have a significant impact on patients' quality of life and often present a significant challenge to manage successfully with conventional therapies alone. The availability of a range of new, targeted therapies for their treatment, most notably the availability of biologic agents, has revolutionised the treatment of both of these conditions.

Secukinumab (Cosentyx), a fully-human monoclonal antibody that acts by selectively binding and neutralising the proinflammatory cytokine IL-17A, is widely used for the treatment of both chronic plaque psoriasis and psoriatic arthritis, with FDA approval for the use in moderate-to-severe chronic plaque psoriasis received in 2015. Despite the important role of agents that target the action of IL-17 in a number of autoimmune inflammatory conditions, their use in inflammatory bowel disease has not been successful, with a number of studies and case reports suggesting that not only is inhibition of IL-17 ineffective for management of Crohn's disease but also that their use may induce or exacerbate the condition.

In this report, we will discuss the case of a 54-year-old female treated with secukinumab for chronic plaque psoriasis and psoriatic arthritis who experienced new onset of symptoms and a diagnosis of inflammatory bowel disease subsequent to treatment with the agent.

## 2. Case

A 54-year-old female with chronic plaque psoriasis and psoriatic arthritis presented with a one-month history of diarrhoea, passing at least five loose motions each day with accompanying urgency, abdominal discomfort, tenesmus, and weight loss of 4 kg. There was no known precipitant and no history of fevers, recent travel, dietary change, or sick contacts. She had no personal history of inflammatory bowel disease, though she had a sister with ulcerative colitis. She was a current smoker and had ceased use of NSAIDs at the onset of symptoms.

Examination was unremarkable. Investigations revealed an elevated faecal calprotectin of 612 *μ*g/g (normal: <50 *μ*g/g). Inflammatory markers were mildly elevated with an erythrocyte sedimentation rate (ESR) of 12 mm/hr and C-reactive protein (CRP) of 26 mg/L. Full blood count, chemistry panel, and iron studies were unremarkable. Faecal microscopy and culture including assessment for ova, cysts, and parasites and multiplex PCR for major enteric pathogens were negative. Serum testing for Strongyloides antibodies was negative. The patient reported a single positive faecal occult blood test that had been performed by her general practitioner.

Given these findings and the patient's ongoing symptoms, she was referred for urgent colonoscopy. This revealed mild-to-moderate patchy ileal inflammation with ulcers, with a normal colon. The impression of the reporting gastroenterologist was that this was consistent with Crohn's disease, possibly induced by secukinumab, and suggested consideration of cessation of the drug.

The patient had been commenced on secukinumab by her rheumatologist fourteen months prior to the onset of symptoms as management for both psoriatic arthritis and chronic plaque psoriasis. She had previously taken methotrexate which was ceased approximately two months after commencing the biologic agent.

Secukinumab was subsequently ceased, and she remained off a biologic agent for two months until worsening of both her chronic plaque psoriasis and psoriatic arthritis necessitated treatment. During this two-month period, her bowel symptoms improved markedly. The decision was made to commence on a biologic agent targeting the p19 subunit of IL-23 and to avoid the use of another agent targeting IL-17, given the diagnosis of inflammatory bowel disease.

She was commenced on tildrakizumab (Ilumya), a monoclonal antibody that selectively binds to the p19 subunit of IL-23, and noted significant improvement in not only her chronic plaque psoriasis and psoriatic arthritis but also complete resolution of her symptoms of inflammatory bowel disease and normalisation of her inflammatory markers. She has remained on this agent for nine-months to date and has reported no adverse events.

## 3. Discussion

Chronic plaque psoriasis and psoriatic arthritis are common immune-mediated conditions, both of which result in significant impairment of a patient's quality of life. Chronic plaque psoriasis is a common inflammatory condition of the skin, affecting 1-2% of the population [1]. It is characterised by the presence of erythematous, thickened, scaly plaques most commonly of the flexor surfaces of the elbows and knees, but may also be seen in the scalp, groin, nails, or palmoplantar surfaces. With a population prevalence of 1%, psoriatic arthritis is characterised by the presence of peripheral and axial arthritis, dactylitis, and enthesitis and is present in up to 30% of patients with chronic plaque psoriasis, most commonly in those with nail psoriasis [2]. The development of targeted biologic therapies for the treatment of chronic plaque psoriasis and psoriatic arthritis has revolutionised clinical practice and provided significant improvements to the quality of life of those who suffer from them when compared to treatment with conventional therapies.

### 3.1. Psoriasis and Inflammatory Bowel Disease

Patients with psoriasis are at a significantly higher risk of experiencing at least one other immune-mediated inflammatory disease (IMID) when compared to the general population [4]. A study of 10,923 patients assessed the rates of IMIDs in patients with psoriasis and compared this to 109,230 general population controls [4]. They reviewed the rates of these conditions before and after psoriasis was diagnosed. They reported that 20% of patients with psoriasis had at least one IMID prior to diagnosis, and 14.7% were found to have an IMID after diagnosis, representing a five-fold increased risk of IMIDs compared to general population controls. Crohn's disease was identified in 1% of patients prior to diagnosis of psoriasis and in 0.2% after diagnosis. Ulcerative colitis was identified in 0.8% of patients prior to diagnosis of psoriasis and in 0.3% after diagnosis. This data suggests that not only will a significant proportion of patients experience comorbid psoriasis and inflammatory bowel disease but also that it is more common for the inflammatory bowel disease to be present at the onset of psoriatic symptoms than for it to develop after a diagnosis of psoriasis.

The association between psoriasis and inflammatory bowel disease is well established and was first reported in 1968, with an epidemiological study suggesting a higher prevalence of psoriasis in first-degree relatives of patients with Crohn's disease [5]. More recently, a Swiss cohort study of 950 patients published in 2011 [6], which included 580 patients with Crohn's disease and 370 patients with ulcerative colitis, assessed the frequency of extraintestinal manifestations. 2% of the patients with Crohn's disease also suffered from psoriasis, and 1% of the patients with ulcerative colitis were also diagnosed with psoriasis. A Danish population study of 5,554,100 individuals published in 2016 investigated the association between Crohn's disease and ulcerative colitis in patients with psoriasis over a period from 1997 to 2012 [7]. The lifetime prevalence of Crohn's disease and ulcerative colitis in this population was higher in those diagnosed with psoriasis, with 0.65% of individuals with psoriasis developing Crohn's disease, compared to 0.35% of the general population, and 1.41% of individuals diagnosed with psoriasis developing ulcerative colitis, compared to 0.81% of the general population. The mean time to diagnosis from the onset of psoriasis was 3.9 years for Crohn's disease and 4.3 years for ulcerative colitis. It is important to note that the study excluded patients with Crohn's disease, ulcerative colitis, or psoriasis prior to the start of the study period, so it did not discuss the rates of ulcerative colitis and Crohn's disease prior to diagnosis of psoriasis.

Like psoriasis, rates of inflammatory bowel disease are higher in family members of those diagnosed with the condition [8]. A Danish population study published in 2015 assessed familial risk for inflammatory bowel disease [8]. They reported that up to 12% of patients with Crohn's disease and 9% of patients with ulcerative colitis had a family history of inflammatory bowel disease. Their results showed an eight-fold increase in the risk of Crohn's disease in first-degree relatives of those with Crohn's disease and a four-fold increase in ulcerative colitis in first-degree relatives of those with ulcerative colitis when compared to families with no history of inflammatory bowel disease. This risk was also increased in second- and third-degree relatives, though to a lesser extent. They also noted that a history of a family member with ulcerative colitis corresponded to an increased risk of a diagnosis of Crohn's disease and vice versa.

From this, it is clear that taking comprehensive history, including assessment of the personal and family history of inflammatory bowel disease, and other IMIDs, is of great importance and should help to guide the selection of the most appropriate management options for patients with psoriasis.

### 3.2. IL-17 Induced Inflammatory Bowel Disease–What is the Evidence?

Secukinumab is a fully-human monoclonal antibody that acts by selectively binding and neutralising the proinflammatory cytokine IL-17A [9]. IL-17 is known to play a central role in the pathophysiology underpinning a number of immune-mediated conditions including chronic plaque psoriasis, psoriatic arthritis, multiple sclerosis, and ankylosing spondylitis [9], and as such, secukinumab has been investigated and utilised extensively for range of these conditions.

In its phase 3 trial for chronic plaque psoriasis “ERASURE,” which compared the safety and efficacy of secukinumab delivered by subcutaneous injection at doses of 300 mg and 150 mg to placebo, the percentage of participants who demonstrated a 75% reduction in their Psoriasis Area Severity Index (PASI) at week 12 was 81.6% for 300 mg and 71.6% for 150 mg, compared to 4.5% of patients randomised to placebo, and the percentage of patients achieving an Investigator Global Assessment score of 0 or 1 (clear or almost clear) was 65.3% for 300 mg, 51.2% for 150 mg, and 2.4% in the placebo group [9]. Further to this, secukinumab showed superior efficacy to both entanercept, a fusion protein targeting tumor necrosis factor (TNF), and ustekinumab, a monoclonal antibody targeting the shared p40 subunit of IL-12 and IL-23, in the phase 3 FIXTURE and CLEAR clinical trials, respectively [9, 10].

The FUTURE 1 and FUTURE 2 trials investigated the efficacy of secukinumab in patients with active psoriatic arthritis, with both phase 3 trials reporting statistically significant improvements in a number of domains including signs and symptoms of psoriatic arthritis, numbers of joints actively involved, function, radiographic disease progression, and in patient and physician global assessments in those treated with secukinumab at doses of 300 mg and 150 mg versus placebo, with the response to 75 mg of secukinumab not significantly different to placebo in the FUTURE 2 trial [2, 11].

As a class, agents that target IL-17 have shown significant promise for a range of autoimmune inflammatory conditions. Other agents in this class include ixekizumab (Taltz), a humanised monoclonal antibody targeting IL-17A, brodalumab (Siliq), a human monoclonal antibody targeting the IL-17 receptor A, and bimekizumab, a humanised monoclonal antibody targeting IL-17A and IL-17F which is in phase 3 clinical trials at the time of publication [12].

The role of agents that target IL-17 in the management of inflammatory bowel disease remains controversial. A number of case reports describing new-onset Crohn's disease and ulcerative colitis in those treated with secukinumab have been published [13–17], and a phase II proof-of-concept trial to assess the safety and efficacy of secukinumab in 59 patients with moderate-to-severe Crohn's disease revealed that it was not only ineffective, with 15.4% of patients assigned to active treatment experiencing worsening of symptoms, but also that there was an increased rate of adverse events and serious adverse events in the treatment group when compared to placebo [18].

A review article by Schreiber et al. [19] investigated the incidence rates of inflammatory bowel disease in patients enrolled on 21 clinical trials of secukinumab for the treatment of psoriasis, psoriatic arthritis, and ankylosing spondylitis, with a pooled analysis of 7355 patients and a cumulative exposure time of 16,226.9 patient-years. For these 7355 patients, they report 41 cases of inflammatory bowel disease. In the studies specific to psoriasis, there were 5 cases of Crohn's disease, 14 cases of ulcerative colitis, and 1 “unclassified” case, with 14 of these representing new onset of disease. In the studies specific to psoriatic arthritis, they report three cases each of Crohn's disease and ulcerative colitis and two “unclassified” cases, with seven of these representing new onset disease. The analyses for ankylosing spondylitis revealed eight cases of Crohn's disease, four cases of ulcerative colitis, and one “unclassified” case with nine representing new onset of disease. For comparison, among the placebo groups, there was one case each of Crohn's disease and ulcerative colitis in the 2877 patients randomised to placebo in studies for psoriasis, two cases of Crohn's disease, and one of ulcerative colitis in the 394 patients randomised to placebo in studies for ankylosing spondylitis and none in the 703 patients randomised to placebo in studies for psoriatic arthritis. The occurrence of inflammatory bowel disease has also been described for both ixekizumab and brodalumab [19].

The pathophysiology of inflammatory bowel disease is complex and multifactorial and is thought to result from abnormal immune responses to microorganisms of the intestinal flora secondary to environmental exposures in genetically susceptible individuals, leading to chronic inflammation of the intestinal tract [20, 21]. The exact mechanism by which agents that target IL-17 promote the development or exacerbation of inflammatory bowel disease and, indeed, the role of IL-17 in inflammatory bowel disease remains poorly defined, though numerous theories exist.

A study by Maxwell et al. [22] used an Abcb1a^−/−^ mouse model of colitis to investigate the roles of IL-17A and IL-17RA. They noted exacerbation of colitis secondary to inhibition of IL-17A and IL-17RA which appeared to be facilitated by a number of factors. They noted a reduction in neutrophil aggregation with IL-17 inhibition, thought to be secondary to inhibition of IL-17A-induced production of chemokines which function as neutrophil chemoattractants. This lack of neutrophil aggregation was significant, as it was thought to allow increased bacterial invasion and dispersal. They also reported increased production of INF-y^+^IL-17^+^ double positive effector cells, which was again significant as it may lead to predominance of effector T-cells over regulatory T-cells, exacerbating underlying inflammation. They noted an increase in intestinal permeability in IL-17RA inhibition and breakdown of the intestinal epithelial barrier. Finally, they also reported that IL-17RA inhibition led to a reduction in the expression of gut antimicrobial peptides, facilitating enhanced microbial persistence and invasion. IL-17 is also known to play an essential role in immunity against yeast, with studies suggesting that overgrowth of *Candida albicans* secondary to IL-17 blockade may trigger or worsen inflammation of the gastrointestinal tract [23].

### 3.3. The Role of Novel Biologic Agents Targeting the p19 Subunit of IL-23

The use of agents targeting TNF has revolutionised the treatment of inflammatory bowel disease over the past 15 years [24]. Despite this, approximately one-third of patients with Crohn's disease are classed as primary nonresponders to them, and in up to a further 40% of patients, efficacy is lost over time as a result of development of immunogenicity, rapid clearance of the drug, or due to development of adverse events necessitating cessation of the treatment [25]. This highlighted the requirement for development of more effective therapies for the condition. Agents that target IL-23, either via the shared p40 subunit of IL-12 and IL-23 or more selective agents targeting the p19 subunit of IL-23, have shown promise for the treatment of inflammatory bowel disease, and similarly, a number of these agents have shown significant benefits or remain under investigation for their role in treatment of chronic plaque psoriasis and psoriatic arthritis.

Ustekinumab, a monoclonal IgG1 antibody targeting the shared p40 subunit of IL-12 and IL-23, is approved for use in the treatment of moderate-to-severely active Crohn's disease with its efficacy demonstrated in the UNITI-1, UNITI-2, and IM-UNITI studies [26]. Though its safety profile has been characterised extensively through its trials in psoriasis, there have been notable concerns regarding the potential for malignancy and reduction in host immune defences against pathogens by blocking IL-12-mediated Th1 responses [24, 25], and the utility of blockade of IL-12 in the management of Crohn's disease is disputed [24].

Agents currently under investigation for management of Crohn's disease that selectively target the p19 subunit of IL-23 include brazikumab, guselkumab, mirikizumab, risankizumab, and tildrakizumab [25].

The phase 2a double-blind, placebo-controlled study of brazikumab (known as MEDI2070) in 119 adult patients with moderate-to-severely active Crohn's disease who had failed treatment with agents targeting TNF showed promising results, with 49.2% of patients randomised to MEDI2070 reaching the primary end-point (a 100-point decrease in Crohn's disease activity scores or clinical remission as defined by a Crohn's disease activity score of less than 150) at week eight, compared to 26.7% of patients receiving placebo [27]. At week 24, clinical response was achieved in 53.8% of patients who continued on open label MEDI2070 and in 57.7% of patients who had initially received placebo and, then, subsequently received open label MEDI2070 [27].

Phase 2 studies of risankizumab showed similar promise. A randomised, double-blind, placebo-controlled phase 2 study of 121 adult patients, 93% of whom had been treated with an agent targeting TNF previously and 79% of which had failed treatment with that agent, was conducted [28]. Patients received intravenous (IV) risankizumab at a dose of 200 mg or 600 mg, or placebo, at weeks 0 and 4, and 8. 31% of patients randomised to risankizumab at either dose achieved the primary end point (Crohn's disease activity score of less than 150), compared to 15% in the placebo arm. 24% of patients randomised to the 200 mg arm and 37% of patients randomised to 600 mg risankizumab arm achieved clinical remission. Rates of adverse events were similar between the groups, and there was a higher rate of serious adverse events in the placebo group [28].

An open label extension was performed [29], where patients who failed to achieve “deep remission” (Crohn's disease activity score of less than 150) or endoscopic remission, received open-label IV risankizumab at a dose of 600 mg every four weeks, and those who had achieved remission were “washed out” from the medication for 12 weeks, unless they met the criteria for a “flare” of their disease in this time. Patients remaining in clinical remission at week 26 were then randomised to the “maintenance dosing regimen” of 180 mg by subcutaneous injection every 8 weeks for 26 weeks. They reported an increase in rates of clinical remission in all treatment groups at week 26, with 55% of patients initially randomised to placebo achieving clinical remission at week 26 compared to 18% of this group at week 12, 59% of patients initially randomised to receive 200 mg IV risankizumab achieving clinical remission at week 26 compared to 21% of this group at week 12, and 47% of patients initially randomised to 600 mg IV risankizumab achieving clinical remission at week 26 compared to 26% of this group at week 12. Clinical remission was maintained by 71% of patients at week 52.

Following early promise, a phase II/III, randomised, double-blind, placebo- and active-controlled, parallel-group, multicentre protocol evaluating the efficacy and safety of guselkumab in moderately to severely active Crohn's disease (GALAXI) and a phase III, multicentre, randomised, double-blind, placebo- and active-controlled treat-through study to evaluate the efficacy and safety of study of mirikizumab in patients with moderately to severely active Crohn's disease are currently listed in clinicaltrials.gov as actively recruiting.

A number of the agents under investigation in Crohn's disease are also known to play an important role in the management of chronic plaque psoriasis and psoriatic arthritis. Guselkumab, risankizumab, and tildrakizumab are currently approved for the treatment of moderate-to-severe plaque psoriasis, with mirikizumab in Phase III clinical trials for the indication. The role of these agents in psoriatic arthritis remains under investigation [30], with active phase III trials of guselkumab and risankizumab and a phase II/III trial of tildrakizumab.

## 4. Conclusions

The development and availability of agents that target IL-17 for a range of autoimmune inflammatory conditions, including chronic plaque psoriasis and psoriatic arthritis, have provided an important therapeutic advance, with significant improvements in the patient's symptoms and quality of life. Despite this, agents that target IL-17 have not been successful in the management of inflammatory bowel disease, with use of these agents associated with worsening of or, in some cases, the onset of the condition. It is, therefore, suggested that agents that target IL-17 should be used with caution in patients with comorbid inflammatory bowel disease or symptoms suggestive of it.

The past few years have seen a significant increase in the number of biologic agents approved for use by the FDA. The availability, success, and apparent safety of agents that target the p19 subunit of IL-23 make them an exciting family of agents to consider in patients with chronic plaque psoriasis, especially where there is the suggestion, or diagnosis, of inflammatory bowel disease as a comorbidity. Investigations of their role in psoriatic arthritis are ongoing, with a number of agents showing early promise.

In this report, we have discussed the case of a 54-year-old female who experienced the onset and diagnosis of inflammatory bowel disease while on treatment with an agent that targets IL-17. We have discussed the improvement not only in her symptoms of chronic plaque psoriasis and psoriatic arthritis but also in Crohn's disease, with the use of an agent that targets the p19 subunit of IL-23.

With recent therapeutic advances and the availability of biologic agents that act via a range of mechanisms, it is more important than ever to consider all patient factors and comorbidities and to allow these to guide management choices to ensure the best possible outcome for each individual patient.
